# Assessing the value of PCR assays in oral fluid samples for detecting African swine fever, classical swine fever, and foot-and-mouth disease in U.S. swine

**DOI:** 10.1371/journal.pone.0219532

**Published:** 2019-07-16

**Authors:** Oriana Beemer, Marta Remmenga, Lori Gustafson, Kamina Johnson, David Hsi, Maria Celia Antognoli

**Affiliations:** Surveillance Design and Analysis Unit, Center for Epidemiology and Animal Health, Veterinary Services, Strategy and Policy, Animal and Plant Health Inspection Service, United States Department of Agriculture, Fort Collins, Colorado, United States of America; Panstwowy Instytut Weterynaryjny - Panstwowy Instytut Badawczy w Pulawach, POLAND

## Abstract

**Introduction:**

Oral fluid sampling and testing offers a convenient, unobtrusive mechanism for evaluating the health status of swine, especially grower and finisher swine. This assessment evaluates the potential testing of oral fluid samples with real-time reverse-transcriptase polymerase chain reaction (rRT-PCR) to detect African swine fever, classical swine fever, or foot-and-mouth disease for surveillance during a disease outbreak and early detection in a disease-free setting.

**Methods:**

We used a series of logical arguments, informed assumptions, and a range of parameter values from literature and industry practices to examine the cost and value of information provided by oral fluid sampling and rRT-PCR testing for the swine foreign animal disease surveillance objectives outlined above.

**Results:**

Based on the evaluation, oral fluid testing demonstrated value for both settings evaluated. The greatest value was in an outbreak scenario, where using oral fluids would minimize disruption of animal and farm activities, reduce sample sizes by 23%-40%, and decrease resource requirements relative to current individual animal sampling plans. For an early detection system, sampling every 3 days met the designed prevalence detection threshold with 0.95 probability, but was quite costly.

**Limitations:**

Implementation of oral fluid testing for African swine fever, classical swine fever, or foot-and-mouth disease surveillance is not yet possible due to several limitations and information gaps. The gaps include validation of PCR diagnostic protocols and kits for African swine fever, classical swine fever, or foot-and-mouth disease on swine oral fluid samples; minimal information on test performance in a field setting; detection windows with low virulence strains of some foreign animal disease viruses; and the need for confirmatory testing protocol development.

## Introduction

Oral fluid (OF) samples are an aggregate sample composed of saliva and mucosal transudate, which may contain pathogens, antibodies to pathogens, or both, circulating in the herd. Collecting OF samples offers a convenient and unobtrusive mechanism for evaluating the health status of swine, especially those nearing slaughter and movement to slaughter—namely growing (grower) and market hog (finisher) herds. OF collection takes advantage of natural swine behavior. Samples are collected by hanging cotton ropes in one or more pens in a barn for pigs to chew for 20–30 minutes, causing minimal disruption of animal activities or farm operations [1–4]. The ease of OF sample collection and the ability to obtain disease status information from an entire group with a single sample, combined with its widespread industry usage for endemic diseases, positions OF sampling as a strong methodology for on-farm surveillance of swine pathogens. Producers currently collect and submit OF samples to monitor disease status of barns or premises with minimal training and without added cost, constraints, and biosecurity concerns associated with veterinary or animal health technician farm visits. Diagnostic testing is currently performed on ~30,000 OF samples per year at three high-volume laboratories representing more than 250 premises for domestic pathogens, including swine enteric coronavirus diseases (SECD), porcine reproductive and respiratory syndrome (PRRS), influenza A virus in swine (IAV-S) and others [5, 6].

Testing methodologies have been applied to OF samples collected from swine, including real-time reverse-transcription polymerase chain reaction (rRT-PCR), solid-phase competitive enzyme-linked immunosorbent assay (ELISA), virus isolation, sequencing, and double-antibody sandwich ELISA [7]. Much research has focused on the use of rRT-PCR, both single-plex and multi-plex; single-plex is the method considered for calculations in this evaluation. Diagnostic tests for other sample types have been developed for other diseases, including the foreign animal diseases considered in this evaluation: African swine fever (ASF), classical swine fever (CSF), and foot-and-mouth disease (FMD).

The sensitivity of an rRT-PCR testing protocol applied to OF samples is dependent on the matrix in which the target virus is found, host factors affecting the concentration or composition of the target virus, and non-host factors such as sample quality, which includes sample deterioration due to poor handling or excessive environmental contamination [8]. The matrix with the target virus may contain inhibitors that could prevent the assay from working. The abundance or concentration of the target virus in an OF sample is a host factor that depends on the organism concentration in an individual animal’s oral fluid transudate, the number of animals chewing on the rope at collection time and shedding the virus in saliva, and the timeliness of the rope sampling relative to the virus shedding window. In a field setting, diseased animals may represent only a portion of the population in a particular pen of animals [9, 10]. Available information from a study on pigs experimentally infected with ASF, CSF, or FMD show that infected animals were observed chewing on ropes until severe clinical signs developed, concurrent with or after virus was detectable in OF samples [11]. Regarding the timeliness of rope sampling relative to shedding of virus, it is important to note that for all three diseases considered in this evaluation, the incubation and infectious periods vary depending on the strain of virus and its virulence [12]. From laboratory studies, ASF and FMD are detectable in OF samples before presentation of clinical signs and often concurrent to pyrexia [7, 11, 13, 14]. Detectable levels of CSF virus appear in OF samples at the same time or after presentation of clinical signs [11, 15, 16]. Mostly described for CSF, persistently infected animals may result from vertical transmission in-utero or infection shortly after birth. In such cases, distinct clinical signs may not be present, but animals will continue to shed virus without a detectable antibody response [17, 18]. Some strains of ASF and CSF may present with mild clinical signs indistinguishable from endemic diseases, complicating detection by clinical signs alone [18, 19].

Since the use of rRT-PCR diagnostics has not been validated officially for OF samples in the United States for the detection of ASF, CSF, or FMD, this paper use a series of logical arguments, informed assumptions, and a range of test accuracy parameter values to explore four select hypothetical applications of OF sampling and rRT-PCR testing. These hypothetical applications are used to inform how this sampling modality might be applied for surveillance in an outbreak and for early detection in a disease-free setting as a screening test. All applications require additional refining before fully considering implementation in a regulatory framework. A range of other scenarios and direct comparisons to current surveillance systems were outside the scope of the current study and should be explored through other projects.

## Methods

### Scenarios

We examined the hypothetical application, cost, and cost-effectiveness of information provided by OF sampling tested by rRT-PCR for ASF, CSF, or FMD surveillance in swine for four scenarios. Scenarios 1 and 2 encompass surveillance in disease outbreak situations while scenarios 3 and 4 encompass surveillance activities in the absence of an outbreak. Further, the first three scenarios (1, 2, and 3) were designed using example surveillance characteristics, and scenario 4 was evaluated based on current surveillance efforts for endemic disease (i.e., PRRS). These scenarios were evaluated to help understand how the sample and test type could integrate into surveillance planning. Characteristics used to inform the scenarios are outlined in the assumptions and characteristics section; some were based on current USDA FAD Preparedness and Response Plan documents for FMD and CSF [20, 21].

#### Scenarios 1 and 2

**Outbreak surveillance assuming prevalence detection thresholds of 20% infected pens/barn, 33% infected barns/premises, and 2% premises per zone prevalence**. We evaluated two potential surveillance schemes in response to an ASF, CSF, or FMD outbreak. In both cases, the sampling method could be applied to the zone(s) designated around each infected premises, but would vary based on the location of infected premises and overlap between multiple zones. Surveillance in these two zones was evaluated separately as **scenario 1**–outbreak surveillance to support classification of premises status in a control area (minimum 10km area located directly around an infected premises) and **scenario 2** –outbreak surveillance within the surveillance zone (minimum 10km area that forms a ring around the control area) to show no evidence of infection because of differences in specific surveillance objectives required in these zones (objective evidence that a premises is not infected vs monitoring for disease spread outside the control area). Animal movement testing was not evaluated due to information limitations, the complexity of different movement scenarios, and the high number of movement scenarios.

#### Scenarios 3 and 4

**Early disease detection prior to clinical signs in a disease-free setting**. Two additional scenarios assessed were early disease detection to estimate the sample size needed to meet 0.95 probability of detection, assuming prevalence detection thresholds of 20% pen, 33% barn, and 2% premises levels. We calculated the probability of detecting disease prior to clinical signs using two different testing frequencies of a representative number of OF samples from the U.S. swine population. **Scenario 3** was evaluated with a sampling frequency of every 3 days for detection of at least one infected barn per premises. **Scenario 4** was evaluated with sampling on a weekly basis, aligning with current industry practices used to monitor endemic diseases [2, 5].

### Evaluation approach

Throughout this document, a *sample* is defined as OF collected from one rope from a single pen of 25 animals. A *sampling event* is the compilation of samples collected from a specified unit (a pen, a barn, or a geographic region such as State or country) during a predetermined period of time. *Early detection* is defined as the ability to detect disease with testing prior to detection by clinical signs. Further, *validation* refers to the OIE definition from Chapter 1.1.6 of the Manual of Diagnostic Tests and Vaccines for Terrestrial Animals: “Validation is a process that determines the fitness of an assay, which has been properly developed, optimised and standardised, for an intended purpose” and as outlined in the validation pathways [8]. Unless otherwise stated, for the remainder of this assessment, *testing of OF samples* refers to the three foreign animal diseases listed above.

The value of the information provided by OF sampling was measured by effectiveness, which is defined in this document as reaching an ideal surveillance target (0.95 probability of detecting a disease given a specified prevalence detection threshold in a specified time period at the premises level). The surveillance target described the probability of detection that must be met for each scenario considered in the cost-effectiveness evaluation. For scenarios 1 and 2, we evaluated cost-effectiveness to determine the best applications for OF sampling and testing in field settings compared to the currently outlined individual animal testing plans. The cost-effectiveness analysis is readily transferrable to additional pathogens or revised parameter estimates. We conducted a cost analysis for all four scenarios to estimate the total cost for each application of OF sampling and testing.

We examined the sample size required to meet differing surveillance objectives (scenarios 1, 2, and 3). For scenario 4, we calculated the detectable prevalence based on industry’s routine OF sampling efforts for detection of endemic diseases (such as PRRS or SECD). Surveillance sampling requirements are influenced by several features of test performance, including diagnostic test sensitivity presuming a perfect sample (known herd status, optimum timing of sample collection relative to the disease detection window, and optimized test processing), external factors such as pig behavior and environmental impacts on sample quality, and the sampling protocol (initial and follow-up testing to determine confirmed-positive results). The requirements for an effective testing regime are also impacted by temporal accuracy. Temporal accuracy depends on (1) the length of time the pathogen is shed in OFs at detectable levels in the sample and (2) whether sampling placement, frequency, and timeliness successfully target high-risk populations and periods. Finally, the sampling requirements are a function of the number of premises and the number of animals on those premises in the population under surveillance and the population of interest, which in this evaluation depends on the scenario.

In addition to information gaps related to test diagnostic sensitivity and field factors, the optimal sampling and testing frequency with OF samples for ASF, CSF, or FMD has not been fully determined. Test validation is needed, particularly a positive cohort study to determine test diagnostic sensitivity. While data can be extrapolated from laboratory studies on ASF, CSF, and FMD, along with field applications of testing for other endemic diseases, validated test performance in field settings would help to more accurately calculate sample size, false-positive rate, and costs. The testing frequency is dependent on the incubation period of the given ASF, CSF, or FMD strain and the window of detection for the virus in OF samples. As additional data become available regarding diagnostic test parameters, disease distribution, movement patterns, and similar information, sampling design may need to be further refined.

### Assumptions and characteristics

To aid comparison across scenarios, we made several assumptions prior to evaluation to determine effectiveness of the different scenarios.

#### Disease detection window

Based on literature summarized in Table 1, ASF may be detected in OF samples 2–4 days prior to clinical signs, CSF may be detected in OF concurrent to clinical signs, and FMD may be detected in OF samples 1–3 days prior to clinical signs. Note that the specimen types listed are not exhaustive; the list includes those samples that would currently be used in an ASF, CSF, or FMD investigation or are being compared (i.e., oral fluids). There is currently no evidence on how persistently infected animals would relate to ASF or CSF detection in OF samples. Presumably, ASF or CSF persistently infected animals would be detected by OF sampling due to viral shedding without clinical signs; this assumption was used in the evaluation. For the scenarios evaluated, 3 days was assumed to be the number of days the testing could detect disease prior to clinical signs; this value falls within the range of both ASF and FMD. This assumption was also used to estimate how frequently samples would need to be collected to detect disease prior to presentation of clinical signs.

**Table 1:** Timing of first virus detection in various samples and presentation of first clinical signs, in days after exposure to the disease.

Disease	Oral fluids	Oral swabs	Serum/blood	Nasal swabs	Fever	Clinical signs
**ASF** [11, 13, 14]	3 d	5–8 d	5–10 d	5–7 d	5–7 d	5–10 d
**CSF** [11, 15, 16, 22]	5–8 d	3–7 d	2–8 d	---	3–5 d	4–5 d
**FMD** [7, 11]	1 d	2–3 d	1–3 d	2–3 d	1–4 d	2–7 d

[^7^Senthilkumaran et al., 2017; ^11^Grau et al., 2015; ^13^Davies et al., 2017; ^14^Guinat et al., 2014; ^15^Dietze et al., 2016; ^16^Petrini et al., 2017; ^22^Weesendorp et al., 2009]

#### Diagnostic test sensitivity and specificity

Diagnostic sensitivity and specificity ranges for rRT-PCR on OF are shown in Table 2 [7, 11, 23]. To simplify calculations, a follow-up testing protocol was assumed to occur with initial non-negative results to rule out laboratory or test inconsistencies, wherein a non-negative result detected at a National Animal Health Laboratory Network laboratory would be sent to a USDA reference laboratory for follow-up testing [4]. The USDA reference laboratory was assumed to run the same type of rRT-PCR test on the referred OF sample with the same diagnostic specificity. Initiation of a full FAD investigation (to run confirmatory testing) would occur should the follow-up test also give a non-negative result. Oral fluid diagnostic test sensitivity used to determine sample size was conservatively assumed to range from 62% to 95% based on previously published values (Table 2), input from USDA National Veterinary Services Laboratories (NVSL), and near optimal conditions for sample collection and submission. Based on the range of values from the literature and input from USDA NVSL experts, the diagnostic test specificity used for these scenarios was estimated to be as low as 99% and as high as 99.99%.

**Table 2:** Range of oral fluid rRT-PCR diagnostic test sensitivity and diagnostic test specificity.

Virus	Sensitivity	Specificity
African swine fever virus[11]	91%	100%
Classical swine fever virus[11]	62–77%	100%
Foot-and-mouth disease virus[7, 11, 23]	100%	100%
	67–92%	81–95%
	100%	100%

[^7^Senthilkumaran et al., 2017; ^11^Grau et al., 2015; ^23^Vosloo et al., 2015]

#### Premises/animal characteristics

After analyzing data from the National Animal Health Monitoring System and the National Agricultural Statistics Service and consulting with industry experts, we selected two example counties in Iowa to model the large mock swine populations used in this assessment. One example had the highest total swine population (e.g., Sioux County with 1,177,164 swine) and one had the highest density of swine per premises (e.g., Wright County with an average 13,075 swine/operation) [24]. The number of hogs in the example counties was divided by a typical barn size of 1,250 to 1,750 animals (50–70 pens in a barn and 25 swine per pen) to arrive at these estimates [2, 5]. Average number of barns per premises, pigs per pen, and number of premises in a control area or surveillance zone were calculated based on these characteristics for evaluation. Using the informed assumptions for the population information, we estimated that 25 to 50 premises would fall within a hypothetical control area and 75–150 premises would fall within the boundaries of a hypothetical surveillance zone. Based on a 2% prevalence detection threshold and 1,250 animals per barn, individual animal sampling would require at least 157 animals to be sampled per premises [20, 21]. The number of ropes per pen was held constant at one rope per pen [5]. For this assessment, the pen size was assumed to be 25 swine per pen, 50–70 pens per barn, 1,250–1,750 swine per barn, 25–50 premises per control area, and 75–150 premises per surveillance zone. These characteristics are summarized in Table 3.

**Table 3:** Characteristic values held in common across scenarios.

Factor		Value Used
Detection window in OF samples prior to clinical signs[7, 11, 13–16, 22]	ASF	2–4 days
	CSF	0–1 days
	FMD	1–3 days
Prevalence detection threshold[33]	Premises level	2%
	Barn level	33%
	Pen level	20%
Probability of detection		0.95
Diagnostic test sensitivity[7, 11, 23]		62–95%
Diagnostic test specificity[7, 11, 23]		99–99.99%
Pen size[5, 24]		25 head
Barn size[5, 24]	Pens	50–70
	Animals	1250–1750
Barns per premises[5, 24]		3 barns
Premises in 10-km control area[20, 21]		25–50 premises
Premises in 10-km surveillance zone[20, 21]		75–150 premises
Individual animal samples per premises[20, 21]		157
Large scale outbreak length[25]		97 days
OF sampling and testing costs[35–40]		$50/sample
Investigation and confirmatory testing costs[35–40]		$471-540/follow up

[^7^Senthilkumaran et al., 2017; ^9^Vosloo et al., 2015; ^11^Grau et al., 2015; ^13^Davies et al., 2017; ^14^Guinat et al., 2014; ^15^Dietze et al., 2016; ^16^Petrini et al., 2017; ^20^USDA APHIS Veterinary Services, 2014; ^21^USDA APHIS Veterinary Services, 2013; ^22^Weesendorp et al., 2009; ^23^Vosloo et al., 2015; ^24^USDA NASS, 2017; ^25^Delgado et al., 2015; ^33^Davies et al., 2002; ^35^IADDL, 2016; ^36^ISU VDL, 2016; ^37^KSU VDL, 2016; ^38^PADLS, 2016; ^39^UMN VDL, 2016; ^40^USDA APHIS VS FADDL, 2016]

#### Timing and frequency characteristics

Sampling frequencies for scenarios 1 and 2 were taken directly from the current USDA FAD Preparedness and Response Plan documents for CSF and FMD, or modeled after these documents in the case of ASF [20, 21]. In both documents, testing for acute, highly pathogenic strains in the control area should occur “on each premises every 5^th^ day for the duration of the area quarantine or a minimum of 28 days.” In the surveillance zone, a subset of premises is randomly selected from the entire population of premises within the zone for sampling to achieve a 5% prevalence detection threshold at the zone level. Samples from selected premises should occur “once during the first 3-week period of the area quarantine,” then “once during each additional 3-week period of the area quarantine,” for a minimum of two sample collections per surveillance zone. The 2013–2014 USDA documents do not outline the specific frequency or use of aggregate samples for testing, such as OF. Based on previous modeling work performed by Delgado et. al [25] to evaluate laboratory response capacity, one model suggested that a large-scale FMD outbreak could last 97 days. This value was used to calculate the number of sampling events required in an ASF, CSF, or FMD outbreak scenario for one control area and surveillance zone.

Sampling frequency for a national-level early detection system was estimated at every 3 days, the ideal frequency to detect disease prior to clinical signs. For this assessment, we applied susceptible, exposed, infected, and recovered (SEIR) modeling [26–28] with characteristics of a high virulence FMD strain [29, 30]. These models indicated that testing OF samples every 3 days plus monitoring for clinical signs detected disease prior to detection by clinical signs alone more than 95% of the time, while weekly testing of OF samples plus monitoring for clinical signs found disease prior to detection by clinical signs alone 41% of the time. Modeling with characteristics for a moderately virulent Paderborn strain of CSF [31] indicated that weekly testing of OF samples for CSF plus monitoring for clinical signs would provide a detection advantage compared to relying on clinical signs alone for disease detection. It is likely moderate virulence ASF or FMD would follow a similar pattern.

Since sampling every 3 days is an ideal but highly intensive sampling frequency, scenario 4 was included to evaluate what level of detection could be achieved if sampling frequency reflected monitoring practices industry currently uses for endemic disease. Typically, industry production systems submit weekly samples for monitoring [5].

#### Sample size characteristics

We calculated sample sizes in Microsoft Excel using the Cannon formula to account for imperfect diagnostic test sensitivity [32]. For scenario 4, the sample size was calculated based on weekly sampling and the premises/barn size characteristics specified above to determine the probability of disease detection prior to recognition of clinical signs. We evaluated the sampling unit as the barn. Sampling could potentially be reduced through alternative designs, e.g., addressing spatial autocorrelation. However, we focused on conventional approaches for this study.

#### Probability of detection and prevalence levels

The probability of detection given disease is present was set at 0.95 for scenarios 1, 2, and 3 with the goal of establishing a disease detection system. This equates to 95% certainty the surveillance system would have found the disease if present, where presence is defined by the detection threshold (or design prevalence). While a wide range of pen-level, premises-level, and population-level prevalence could be used to design a surveillance system, this project used examples of 20% infected pens per barn, 33% infected barns per premises, and 2% premises per zone/region/country sampled. The 2% premises prevalence with 0.95 probability of detection has been outlined in the FAD Preparation and Response Plan materials [20, 21] and has also been used in FMD outbreaks in the United Kingdom for designing surveillance zone sampling strategies [33]. For scenario 4, the same detection threshold was used to evaluate what probability of detection could be achieved by this scenario.

### Cost assumptions and characteristics

A cost analysis presents the expenses associated with all surveillance activities performed in OF sampling and testing in the four scenarios. In our cost analysis, we did not estimate expenses resulting from any interruption to trade or business continuity but instead focused on the cost of OF sample and testing surveillance activities. Additionally, we calculated cost-effectiveness for scenarios 1 and 2, for comparison to the current outbreak surveillance sample and testing plan.

#### Surveillance sample initial and follow-up testing costs

The cost of testing the target number of samples is the per-sample cost multiplied by the sample size calculated for the determined objective. The cost per OF sample was estimated as $5.56 per collection kit [34], $15 shipping and handling (assumption), and laboratory PCR testing fees of $32 to $35 per sample including possible accession fees [35–39]. The total estimated cost per OF sample tested ranges from $55.56 to $62.56. For simplicity, this evaluation used an estimated $50 per OF sample tested to cover the initial test expenses related to collection, shipping, and laboratory fees based on the assumption that a higher volume of testing could result in a lower per-sample cost. This is summarized in Table 3. Samples with non-negative test results are shipped to NVSL for a follow-up test, which is assumed to have the same $50 cost.

#### ASF, CSF, or FMD investigation and confirmatory testing costs

The per-sample cost is $71 for CSF confirmatory testing following the current testing protocol (Ab ELISA, immunoperoxidase, and immunoperoxidase virus neutralization) [21, 40]. The total estimated cost per sample collected and tested for ASF or FMD is approximately $140 following the current confirmatory testing protocol using RT-PCR and virus isolation testing costs [20, 40, 41]. For the scenarios in this paper, if an oral fluids sample has a non-negative result after follow-up testing at NVSL, an FAD investigation will be initiated and confirmatory testing will be performed at the NVSL Foreign Animal Disease Diagnostic Laboratory. The estimated value of 8 hours of VS employee time is $400 to complete an FAD investigation and ship samples to laboratories for each premises. As a result, the total cost of a false positive would be a minimum of $471 (CSF) to $540 (ASF or FMD), assuming that samples are collected from one animal during an epidemiologic investigation. These costs exclude any domestic business or trade impacts. These characteristics are summarized in Table 3.

#### Cost analysis calculations

Several dimensions contribute to the calculations for the overall cost range. These include differences in number of oral fluid samples collected because of test sensitivity and specificity ranges, in number of sampling events, in testing cost of individual samples, and in number of epidemiologic investigations and samples collected for confirmatory testing. Essentially, the combination of the lowest values for all applicable dimensions is presented as the lowest bound of the overall cost range while the combination of the highest values for all applicable dimensions is presented as the highest bound of the overall cost range.

#### Cost-effectiveness calculations

To calculate the total cost to acquire the same disease status information for scenarios 1 and 2, the following information is needed: 1) the number of oral fluid samples required; 2) the number of samples required to use the current testing protocol; and 3) their respective costs. Cost-effectiveness is calculated by dividing the cost of a surveillance option by its associated cost; a lower cost-effectiveness ratio is preferred as it is considered more cost-effective. Cost savings is the difference between the costs of the two different testing protocols for initial testing (not follow-up or confirmatory testing).

## Results

Table 4 summarizes the evaluation results of each scenario.

**Table 4:** Summary results for scenarios 1, 2, and 3 including sample numbers and costs. Summary results for scenario 4 include the prevalence that can be detected using common industry practices.

Scenario	1	2	3	4
Short description of scenarios and objectives	Outbreak response, assess control area disease status every 5 days for up to a 97-day outbreak	Outbreak response, assess surveillance zone disease status every 21 days for up to a 97-day outbreak	Active national surveillance for early detection, sampling every 3 days for one year	Active national surveillance, sampling weekly for one year
Total sample size per premises	720 up to 1,260^1^	72 up to 315^1^	4,392 to 7,686^1^	520 to 832^2^
Pen-level prevalence that can be detected with 0.95 probability	20% (by design)	20% (by design)	20% (by design)	46 to 100%^3^
Cost of total sample size per premises	$36,000 to $63,000	$3,600 to $15,750	$219,600 to $384,300	$26,000 to $41,600
Number of premises	25 to 50^4^	61 to 81^5^	156 to 240^5^	268^2^
Premises-level prevalence that can be detected with 0.95 probability	1 in N (premises in control area)	2% (by design)	2% (by design)	4.1% to 8.4%^6^
Total sample size per scenario	Up to 18,000 to 63,000	4,392 up to 25,515	685,152 to 1,884,640	181,000^2^
Cost of total sample size per scenario	$900,000 to $3,150,000	$219,600 to $1,275,750	$34,257,600 to $92,232,000	$9,050,000
Total cost including follow up costs for non-negative samples^7^	$900,000 to $3,150,000	$219,650 to $1,290,170	$34,261,050 to $93,254,250	$9,050,950 to $9,150,760

^1^ Range of samples represents the full range from previously outlined low and high sensitivity values and the sampling required over time to meet the scenario objectives.

^2^ Based on average number of rope samples submitted per premises (10 to 16) and average number of premises submitting to large swine veterinary diagnostic laboratories in the U.S.

^3^Assumes equal distribution of rope samples among 3 barns per premises and the full range of sensitivity values.

^4^ Sampling all premises in the control area.

^5^Alternatively, a lower level of sampling per farm and sampling more farms could give the same prevalence detection threshold at the premises level, but reduce prevalence detection threshold at the pen-level.

^6^Calculated assuming the among pen prevalence threshold that needs to be detected on each premises is 20% within a three day period to compare results to scenario 3.

^7^Sum of cost of total sample size per scenario for initial testing and range of total costs for follow up and confirmatory testing for initial samples with non-negative results

### Scenarios 1 and 2: ASF, CSF, or FMD surveillance in outbreak settings

In Scenarios 1 and 2 for outbreak surveillance, we assumed prevalence detection thresholds of 20% infected pens/barn, 33% infected barns/premises, and 2% premises per zone prevalence in a control area or surveillance zone (Table 4).

The USDA FAD Preparedness and Response Plan documents for FMD and CSF both indicate that a premises designation within the control area requires objective information on disease status before a premises may be considered for a different designation (i.e., to move from “at-risk” to “monitored”). Because OF samples provide aggregate information for the premises as a whole within either a control area or surveillance zone, the number of samples required every 5 days for the duration of the outbreak would be much lower for OF sampling (36–63 samples per premises) than for individual animal sampling (157 samples per premises) to achieve 0.95 detection probability at the 20% prevalence detection threshold. This difference translates to requiring OF sample levels of 23% to 40% of the individual animal sampling levels to acquire the same disease status information. Oral fluid sampling costs per premises (with three barns) per control area (scenario 1) range from $36,000 to $63,000 with a cost savings of $194,940 to $384,600, which is approximately 84% to 86% lower in cost. These estimates only include the cost of initial OF testing compared to using the current testing protocol, plus the cost of an epidemiologic investigation as a proxy for the cost to collect individual samples. These do not include any follow-up testing or FAD investigations started because of a non-negative test result. Oral fluid sampling costs per premises (with 3 barns) per surveillance zone (scenario 2) range from $3,600 to $15,750 with a cost savings from $19,494 to $96,150, which is also approximately 84% to 86% lower in cost. When applied to testing multiple premises within a control area or surveillance zone, additional costs may be incurred to evaluate non-negative test results depending on the strategy for handling false positives during an outbreak.

### Scenarios 3 and 4: Early disease detection prior to clinical signs (Table 4)

#### Scenario 3

To achieve a goal of early detection through OF sampling and testing at a 0.95 probability of detection, premises level sampling would have to be performed approximately every 3 days for diseases such as ASF or FMD, given the short window of detection in OF samples prior to presentation of clinical signs. Depending on the test sensitivity, the number of samples required could range from 685,152 to 1,844,640 at a cost of between $34,257,600 and $92,232,000. With a test specificity ranging from 99 to 99.99%, the number of false positives to undergo follow-up testing would be expected to range from 69 to 18,447 with 0 to 185 additional tests for FAD investigations resulting in a combined cost of $3,450 to $1,022,250 for follow-up and confirmatory testing.

#### Scenario 4

Currently, collection of OF samples for domestic disease testing is generally performed weekly, with an average of 10–16 samples per premises [5]. The average number of samples currently submitted to two large swine veterinary diagnostic laboratories per year is approximately 181,000, which we estimate costs $9,050,000. With a test specificity ranging from 99 to 99.99%, the number of false positives needing follow-up tests would be expected to range from 19 to 1,810 with 0 to 19 additional tests for FAD investigations resulting in a combined cost of $950 to $100,760. Scenario 4 requires 11% to 12% of the OF samples required for scenario 3, but can only detect 4% to 8% prevalence instead of the target of 2% premises-level prevalence every 3 days. The cost savings following scenario 4 range from $193,600 to $342,700, which is approximately 88% to 89% lower than scenario 3.^3^

With the lower number of samples per barn, the ability to detect an infected premises is decreased compared to scenario 3. If the diagnostic test sensitivity is at the low end of the assumed range (0.62), a sample size of 10 ropes only provides a probability of 0.51 of detecting at least one infected premises when 2% of the premises are infected, with 20% of the pens infected per premises. If the test sensitivity is at the high end of the assumed range (0.95), a sample size of 16 ropes provides a probability of 0.76 of detecting at least one infected premises when 2% of the premises are infected, with 20% of the pens infected per premises.

## Discussion and conclusion

This evaluation compared various scenarios for swine OF sampling to test for ASF, CSF, or FMD by rRT-PCR. Scenarios included an assessment of premises disease status during an outbreak setting in the control area and surveillance zone, and an early detection active surveillance system. Understanding the cost of effective surveillance schemes using OF sampling and testing for ASF, CSF, or FMD swine surveillance will assist in selecting best use applications and prioritizing future test validation and surveillance design development needs. Many characteristics were derived from available literature and data sources, but we used assumptions to overcome some information gaps. For all scenarios except scenario 4, the prevalence detection threshold and detection probabilities were set at an ideal level. A lower level of prevalence detection threshold or detection probability using OF may still provide beneficial information at a lower total cost than current testing protocols.

Of note, the diagnostic test sensitivity values on OF samples could be outside the range of values used in this evaluation. Lower published rRT-PCR sensitivity values can be found for influenza A virus in swine [42] and other tests used with endemic disease monitoring [43]. Additionally, this evaluation did not include an adjustment factor for field impacts from animal behavior, sample dilution, degradation, handling, and submission logistics. These factors could also lead to lower test parameter values. Since hypothetical test characteristics were used, the actual test utility will largely depend on the results of test validation studies, which could further guide scenario development, comparison, and drawing further conclusions.

### Scenarios 1 and 2

Outbreak surveillance assuming prevalence detection thresholds of 20% infected pens/barn, 33% infected barns/premises, and 2% of premises per zone prevalence in a control area or surveillance zone. During an outbreak response, OF sample testing offers many advantages compared to individual animal sampling, including minimal disruption of animal and farm activities and a reduction in the number of samples required, thus reducing testing costs. In both scenarios 1 and 2, the cost-effectiveness ratio of aggregate OF samples is lower than individual animal sampling. Additionally, OF sample testing could decrease resource utilization. Given basic training, producers can collect and submit OF samples to emergency response officials at the premises line of separation; emergency response officials would not need to enter the farm. This approach could decrease resource costs associated with personal protective equipment, waste disposal, and personnel downtime between farms, minimizing potential contamination between premises by eliminating animal contact. Depending on the disease, OF sample testing may detect viral presence prior to, or concurrent with, clinical signs. This information could assist policymakers and incident commanders with more timely, cost-effective information upon which to base decisions. Because poor sample handling technique can decrease test sensitivity, a certification/training program on proper sample collection and packaging should be implemented for farm personnel, accredited veterinarians, veterinary paraprofessionals, and response officials. Such a program, outlined in the Secure Pork Supply Plan for Active Observational Surveillance [44], would assist with outbreak preparedness and should lead to higher quality samples.

Beyond the logistical considerations of using OF testing during an ASF, CSF, or FMD outbreak, sampling is also naturally targeted both spatially and temporally, and the duration of sampling is limited by a known period of exposure and a foreseeable end-date (the end of the quarantine). Oral fluid sampling may be highly efficacious in this scenario if the benefit of preventing disease spread is greater than the cost of a short but intensive sampling scheme. Presuming use during an outbreak to assist in prioritization of incident response activities (e.g., to prioritize order of formal surveillance or inspection visits), the consequences of a false negative or positive are similarly constrained. Laboratory capacity could limit the application of any novel testing strategy if the number of samples generated by an outbreak exceeds laboratory capabilities. Regardless of the scale of an actual outbreak, OF collection is expected to generate fewer samples compared with individual animal sampling, thus decreasing testing costs and making more effective use of laboratory capacity. Once test validation information is known, further exploration of potential animal movement and continuity of business scenarios would also be informative.

### Scenarios 3 and 4: Early disease detection prior to clinical signs

#### Scenario 3

The early detection scenario was designed with a 0.95 probability of detecting at least one infected premises prior to clinical signs when infection is at 20% of a pen, 33% of a barn, and 2% among all premises. The scenario assumed sample collection occurred approximately every 3 days to detect disease prior to clinical signs, based on the window of detection in OF samples for ASF and FMD. This scenario could be modified to lower levels of sampling for different goals, which would still be useful in potentially detecting disease earlier than with passive surveillance alone while lowering overall cost, which was partially explored in scenario 4. The windows of detection for ASF, CSF, and FMD were extrapolated from laboratory experiments using high virulence strains, leading to rapid clinical progression of disease. The detection window with OF samples and progression of clinical signs are not as thoroughly documented when less virulent strains of ASF, CSF, or FMD are studied. Given the currently available data on highly virulent CSF detection in OF samples, it is unlikely this sampling and testing scenario fits a CSF rapid detection purpose because clinical signs, if seen at all, present at the same time as detectable virus in OF samples. If viral shedding in OFs occurs before detectable clinical signs for ASF, CSF, or FMD are observed, the frequency of sampling could be decreased.

For moderate or low virulence diseases that present with non-specific signs (i.e., CSF or FMD), OF sampling and testing may prove more valuable. The SEIR modelling performed for this assessment, with characteristics for a moderately virulent strain of CSF (Paderborn strain), indicated there may be an advantage to weekly OF sampling and testing for CSF. It is likely ASF and FMD would follow a similar pattern if a low virulence strain or chronic form of the disease were introduced. However, given the results of this evaluation, the potential application of OF sampling for active surveillance as a large-scale early detection system would be costly.

#### Scenario 4

This early detection scenario does not achieve a 0.95 probability of detecting at least one infected premises prior to clinical signs for the range of test sensitivities evaluated using common industry sample submission practices. A higher number of samples submitted per barn or sampling a greater number of premises could achieve 0.95 probability of detection. The scenario presented assumed that each premises collected samples weekly and that the submissions were distributed throughout a given week to temporally space submissions. This scenario represents a lower level of sampling than scenario 3, and may still detect disease earlier than passive surveillance alone with a lower overall cost. The greatest advantage would likely be realized for diseases with moderate or mild clinical signs, which can be difficult to detect or differentiate from other diseases with clinical signs alone (i.e., CSF or FMD). The cost-effectiveness for scenarios 3 and 4 could be compared to each other; however, the decreased costs associated with scenario 4 relative to 3 must be carefully balanced with the decreased level of detection capability demonstrated in these two scenarios.

Oral fluids represent a potentially valuable sample source for many different scenarios related to current individual animal ASF, CSF, and FMD testing. A benefit common to the scenarios is that OF sample testing for surveillance could contribute to emergency preparedness and response efforts directed toward ASF, CSF, or FMD in swine. Of the scenarios evaluated, OF sample testing may be most valuable in an outbreak setting, where the use of aggregate screening samples would minimize the disruption of animal and farm activities, reduce sample sizes, and decrease resource requirements compared to individual animal sampling regimens. Immediate broad implementation is not possible due to several limitations and information gaps. These include: (1) USDA validation of OF sample diagnostics for ASF, CSF, and FMD in swine is still underway, (2) information on test performance in a field setting is lacking, and (3) confirmatory testing of suspected false-positive OF sample findings may demand time-intensive or costly follow-up testing protocols (e.g., repeat on-farm sampling and/or next generation sequencing).

This evaluation was based on the current understanding of OF sample testing without a USDA-validated test. Test validation efforts through positive and negative cohort studies would improve the broader understanding of OF sample testing by rRT-PCR for ASF, CSF, or FMD in swine. Furthermore, understanding test characteristics in a field setting could be used to refine sample size requirements. These steps would provide reliable test characteristics for more fully evaluating use of this sample and testing method for ASF, CSF, or FMD testing and response plans for swine. This evaluation should be revised if additional studies provide new or updated information about ASF, CSF, or FMD pathogenesis or test characteristics, if industry practices change, or if FAD Preparedness and Response Plan materials are updated.
